# Effects of Melittin Treatment in Cholangitis and Biliary Fibrosis in a Model of Xenobiotic-Induced Cholestasis in Mice

**DOI:** 10.3390/toxins7093372

**Published:** 2015-08-25

**Authors:** Kyung-Hyun Kim, Hyun-Jung Sung, Woo-Ram Lee, Hyun-Jin An, Jung-Yeon Kim, Sok Cheon Pak, Sang-Mi Han, Kwan-Kyu Park

**Affiliations:** 1 Department of Pathology, College of Medicine, Catholic University of Daegu, 3056-6, Daemyung-4-Dong, Nam-gu, Daegu 705-718, Korea; E-Mails: khkim1@cu.ac.kr (K.-H.K.); ewingsar@gmail.com (H.-J.S.); woolamee@cu.ac.kr (W.-R.L.); ahj119@cu.ac.kr (H.-J.A.); kjy1118@cu.ac.kr (J.-Y.K.); 2 School of Biomedical Sciences, Charles Sturt University, Panorama Avenue, Bathurst, NSW 2795, Australia; E-Mail: spak@csu.edu.au; 3 Department of Agricultural Biology, National Academy of Agricultural Science, RDA, 300, Nongsaengmyeong-ro, Wansan-gu, Jeonju-si, Jeollabuk-do 55365, Korea; E-Mail: sangmih@korea.kr

**Keywords:** cholangiopathy, melittin, DDC-fed mice

## Abstract

Cholangiopathy is a chronic immune-mediated disease of the liver, which is characterized by cholangitis, ductular reaction and biliary-type hepatic fibrosis. There is no proven medical therapy that changes the course of the disease. In previous studies, melittin was known for attenuation of hepatic injury, inflammation and hepatic fibrosis. This study investigated whether melittin provides inhibition on cholangitis and biliary fibrosis *in vivo*. Feeding 3,5-diethoxycarbonyl-1,4-dihydrocollidine (DDC) to mice is a well-established animal model to study cholangitis and biliary fibrosis. To investigate the effects of melittin on cholangiopathy, mice were fed with a 0.1% DDC-containing diet with or without melittin treatment for four weeks. Liver morphology, serum markers of liver injury, cholestasis markers for inflammation of liver, the degree of ductular reaction and the degree of liver fibrosis were compared between with or without melittin treatment DDC-fed mice. DDC feeding led to increased serum markers of hepatic injury, ductular reaction, induction of pro-inflammatory cytokines and biliary fibrosis. Interestingly, melittin treatment attenuated hepatic function markers, ductular reaction, the reactive phenotype of cholangiocytes and cholangitis and biliary fibrosis. Our data suggest that melittin treatment can be protective against chronic cholestatic disease in DDC-fed mice. Further studies on the anti-inflammatory capacity of melittin are warranted for targeted therapy in cholangiopathy.

## 1. Introduction

Liver fibrosis refers to a classical outcome of many chronic liver diseases irrespective of the etiology of injury. It is characterized by changes in the composition and quantity of extracellular matrix (ECM) deposits distorting the normal structure by forming fibrotic scars. Failure to degrade the accumulated ECM is a major reason why fibrosis progresses to cirrhosis in liver. Various insults, such as viral infection, drugs or metabolic disorders, contribute to the progression of liver fibrosis. Among them, improper regulation of bile flow (*i.e.*, cholestasis), which causes hepatic inflammation and subsequent tissue injury, is one of the main insults for cholangiopathies [1].

As the main cause of liver-related death, cholangiopathies are also the leading cause of liver transplantations in paediatric patients (50%) and the third leading cause in adults (20%) [1,2]. Cholangiopathies, such as primary sclerosing cholangitis (PSC), primary biliary cirrhosis (PBC) and drug-induced bile duct damage, may result in cholestasis, which is characterized by the loss of cholangiocytes through necrosis by apoptosis [3]. Cholangiocyte proliferation can occur during cholangiopathies, resulting in the formation of new side branches to ducts in an effort to regain function and portal/periportal inflammation [4,5].

Cholangiocyte proliferation is described as the expanded population of epithelial cells at the interface of the biliary tree, which refers to the proliferation of pre-existing ductules, progenitor cell activation and the appearance of intermediate hepatocytes [6,7]. The ability of cholangiocytes to proliferate is important in many different human pathological conditions, such as the regaining of proper liver function and the remodelling of biliary cirrhosis in chronic cholestatic conditions.

Lately, 3,5-diethoxycarbonyl-1,4-dihydrocollidine (DDC)-fed mice have been suggested to be used as a novel model for sclerosing cholangitis and biliary fibrosis. Chronic feeding of DDC in mice causes cholangitis with a pronounced ductular reaction, onionskin-type periductal fibrosis and, finally, biliary fibrosis, reflecting several specific pathological hallmarks of human PSC [8]. This model is therefore especially useful to investigate the mechanisms of chronic cholangiopathies and their consequences, including biliary fibrosis, and to test novel therapeutic approaches, such as melittin, for these diseases [8,9].

Melittin is a cationic, haemolytic peptide that is the major bioactive component in honey bee (*Apis mellifera*) venom, and it has been shown to play a role in attenuating fibrosis in various animal models [10,11]. Previous studies have shown that melittin treatment reduced the expression of inflammatory proteins in inflammatory diseases [12]. These studies are informative, but they are not enough to demonstrate that melittin can prevent the development of the inflammatory molecular mechanisms of sclerosing cholangitis. Therefore, the aim of this work was to determine how melittin could become a profibrogenic control and to characterize the underlying mechanism for this effect. The biological properties of melittin were examined in chronic liver injury using DDC-fed model.

## 2. Results

### 2.1. Effects of Melittin on DDC-Fed Mice

**Figure 1:**
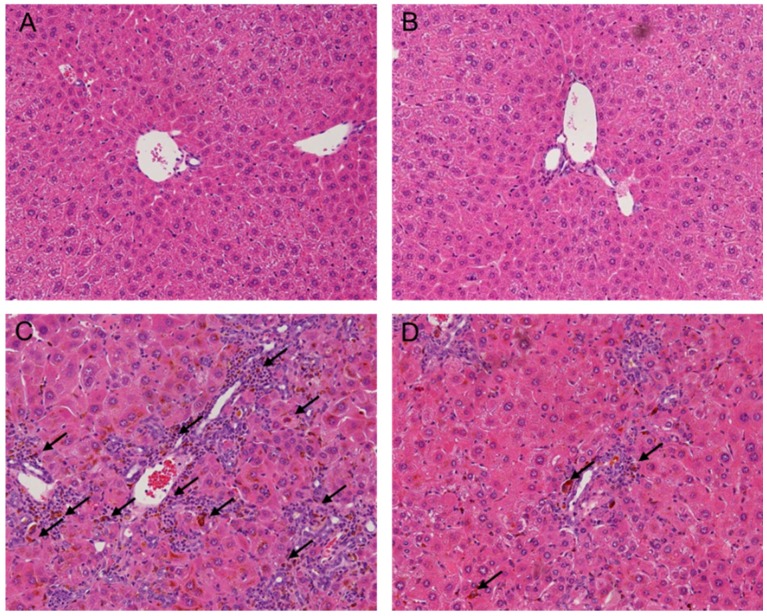
Effect of melittin in DDC-induced liver fibrosis. Hematoxylin and eosin (H&E) staining results show that melittin effectively suppresses inflammation and ductular reaction (arrowheads in (**C**,**D**)) in response of DDC feeding. Representative H&E images from each study group (five mice per group): (**A**) NC, normal control group; (**B**) Mel, melittin (0.1 mg/kg)-treated group with normal diet; (**C**) DDC, 0.1% DDC-supplemented diet group; (**D**) DDC + Mel, melittin (0.1 mg/kg)-treated group with 0.1% DDC-supplemented diet. Magnification ×200.

**Figure 2:**
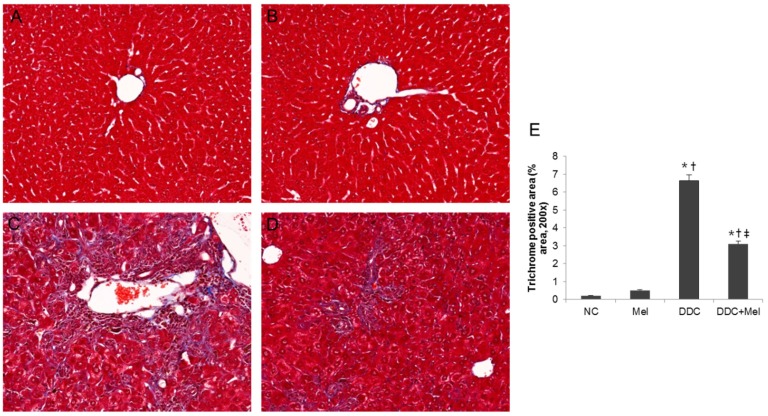
Effect of melittin in DDC-induced liver fibrosis. Masson’s trichrome staining results show that melittin effectively attenuates ECM remodelling following chronic DDC feeding in mice. Representative trichrome staining images from each study group (five mice per group): (**A**) NC, normal control group; (**B**) Mel, melittin (0.1 mg/kg)-treated group with normal diet; (**C**) DDC, 0.1% DDC-supplemented diet group; (**D**) DDC + Mel, melittin (0.1 mg/kg)-treated group with 0.1% DDC-supplemented diet; magnification ×200; (**E**) morphometric assessment of the trichrome staining positive areas. Results are expressed as the mean ± SE of three independent determinations. * *p* < 0.05 compared to the NC group. **^†^** *p* < 0.05 compared to the Mel group. **^‡^** *p* < 0.05 compared to the DDC group.

**Figure 3:**
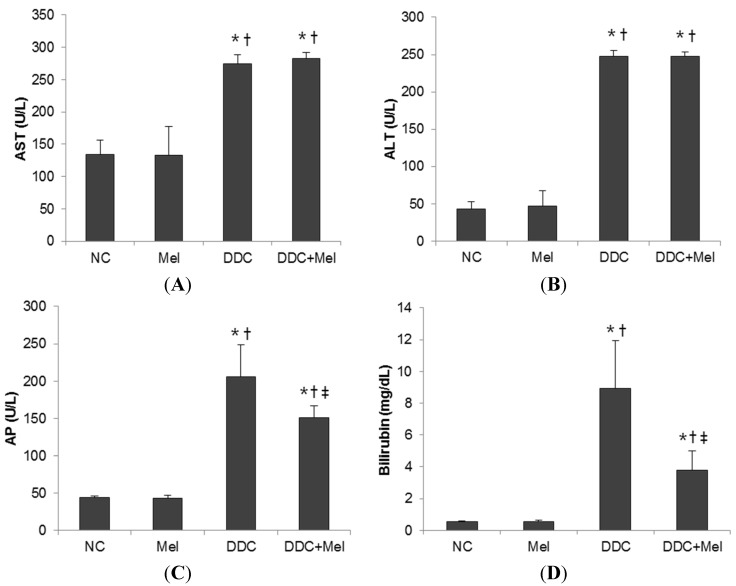
Serum biochemistry of DDC-fed mice. Melittin treatment effectively suppresses serum alkaline phosphatase (AP) and bilirubin, but not aspartate aminotransferase (AST) and alanine transaminase (ALT). (**A**) Serum AST; (**B**) serum ALT; (**C**) serum AP; (**D**) serum bilirubin. NC, normal control group; Mel, melittin (0.1 mg/kg)-treated group with normal diet; DDC, 0.1% DDC-supplemented diet group; DDC + Mel, melittin (0.1 mg/kg)-treated group with 0.1% DDC-supplemented diet. Results are expressed as the mean ± SE of three independent determinations. * *p* < 0.05 compared to the NC group. **^†^** *p* < 0.05 compared to the Mel group. **^‡^** *p* < 0.05 compared to the DDC group.

After four weeks of DDC feeding, liver showed ductules and small bile ducts, which frequently contained pigment plugs (Figure 1). Additionally, pronounced hepatic inflammatory response was observed near bile ducts with predominating neutrophil granulocytes. Furthermore, spontaneous DDC feeding resulted in the deposition of collagen fibres near fibrous septae and expanded bile ducts (Figure 2). These changes were improved by melittin treatment. The DDC + melittin (Mel) group showed the reduction of collagen deposition. DDC feeding also resulted in increased serum AST and ALT levels as an indicator of hepatocyte injury followed by significant elevations of cholestasis parameters of AP and bilirubin. Serum AST and ALT revealed no significant differences between DDC mice and DDC + Mel mice. However, DDC + Mel mice showed significantly lower serum AP and bilirubin levels (Figure 3). These results suggest that melittin treatment effects a decreased susceptibility of cholestasis in DDC-fed mice.

### 2.2. Melittin Treatment Attenuates DDC-Induced Inflammatory Changes

**Figure 4:**
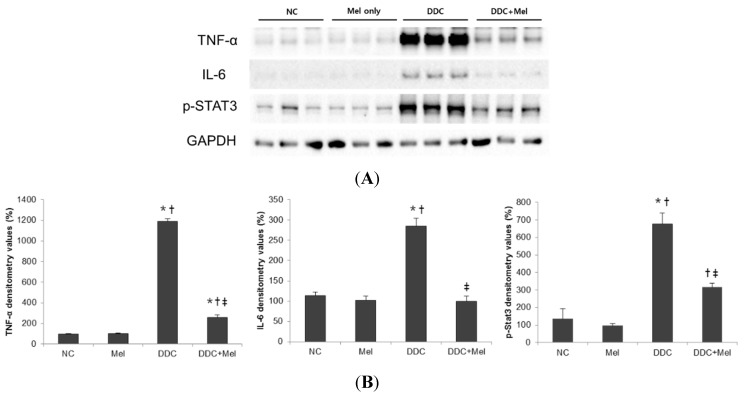
Melittin inhibits pro-inflammatory cytokine expression in DDC-fed mice. (**A**) Western blotting results demonstrated that melittin effectively suppresses the expressions of TNF-α, IL-6 and p-STAT3; (**B**) graphical presentation of the ratio of TNF-α, IL-6 and p-STAT3 to GAPDH in various groups. NC, normal control group; Mel, melittin (0.1 mg/kg)-treated group with normal diet; DDC, 0.1% DDC-supplemented diet group; DDC + Mel, melittin (0.1 mg/kg)-treated group with 0.1% DDC-supplemented diet. Results are expressed as the mean ± SE of three independent determinations. * *p* < 0.05 compared to the NC group. **^†^** *p* < 0.05 compared to the Mel group. **^‡^** *p* < 0.05 compared to the DDC group.

**Figure 5:**
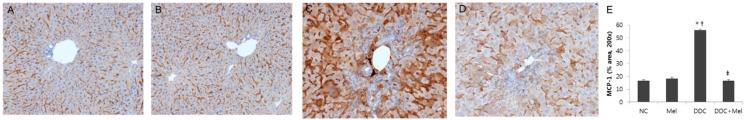
Melittin inhibits inflammatory changes in DDC-fed mice. Immunohistochemical staining results demonstrated that melittin effectively suppresses the expression of MCP-1. Representative immunohistochemical images from each study group (five mice per group) (**A**) NC, normal control group; (**B**) Mel, melittin (0.1 mg/kg)-treated group with normal diet; (**C**) DDC, 0.1% DDC-supplemented diet group; (**D**) DDC + Mel, melittin (0.1 mg/kg)-treated group with 0.1% DDC-supplemented diet; magnification ×200; (**E**) morphometric assessment of the trichrome staining positive areas. Results are expressed as the mean ± SE of three independent determinations. * *p* < 0.05 compared to the NC group. **^†^** *p* < 0.05 compared to the Mel group. **^‡^** *p* < 0.05 compared to the DDC group.

DDC-fed mice demonstrated pronounced hepatic inflammatory response characterized by an increase of infiltrating inflammatory cells near bile ducts (Figure 1C). In contrast, treatment with melittin changed the inflammatory response in portal tracts (Figure 1D). To investigate whether melittin could influence inflammatory changes in DDC-fed mice, pro-inflammatory cytokines were examined in the livers of experimental mice. Pro-inflammatory cytokines, such as TNF-α and IL-6, are key players in eliciting an inflammatory reaction during liver fibrogenesis [13,14]. The expressions of TNF-α and IL-6 were significantly increased in the DDC-fed mice, whereas melittin treatment markedly abrogated this activation (Figure 4). During liver injury, IL-6 activates STAT3 in liver parenchymal and non-parenchymal cells [15,16]. While chronic DDC feeding activated p-STAT3 in the DDC group, the expression level of p-STAT3 was significantly reduced by treatment with melittin. Moreover, the expression level of MCP-1, which promotes liver fibrosis by recruitment of macrophages, was determined by immunohistochemical staining. As shown in Figure 5A,B, MCP-1-positive cells were barely detected in liver sections from the NC and Mel groups. However, MCP-1-positive areas in the DDC group were significantly increased in liver sections, especially near portal tracts (Figure 5C). Compared to the DDC group, treatment with melittin inhibited MCP-1 expression in DDC + Mel liver (Figure 5D). These results indicate that melittin markedly attenuates the levels of pro-inflammatory cytokines during chronic DDC feeding, which may result in the suppression of DDC-induced cholangitis.

### 2.3. Melittin Suppresses Liver Fibrosis in the Livers of DDC-Fed Mice

**Figure 6:**
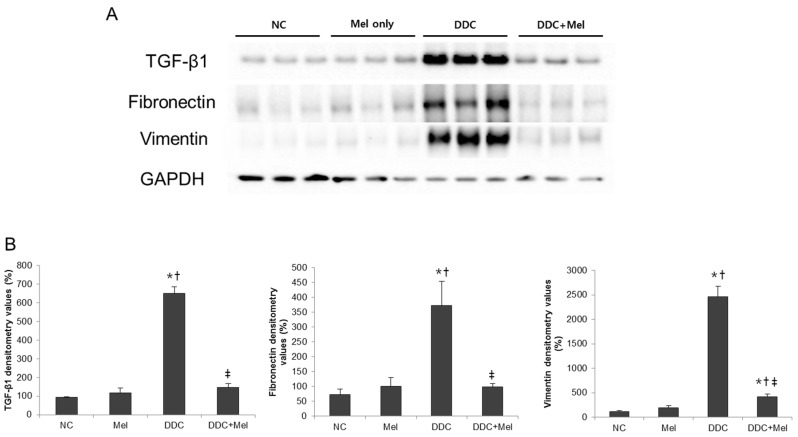
Melittin inhibits fibrosis-related gene expression in DDC-fed mice. (**A**) Western blotting results demonstrated that melittin effectively suppresses the expression of TGF-β1, fibronectin and vimentin; (**B**) graphical presentation of the ratio of TGF-β1, fibronectin and vimentin to GAPDH in various groups. NC, normal control group; Mel, melittin (0.1 mg/kg)-treated group with normal diet; DDC, 0.1% DDC-supplemented diet group; DDC + Mel, melittin (0.1 mg/kg)-treated group with 0.1% DDC-supplemented diet. Results are expressed as the mean ± SE of three independent determinations. * *p* < 0.05 compared to the NC group. **^†^** *p* < 0.05 compared to the Mel group. **^‡^** *p* < 0.05 compared to the DDC group.

**Figure 7:**
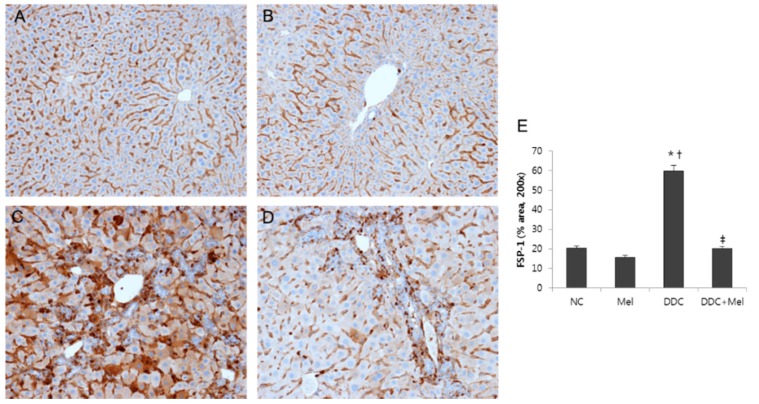
Melittin inhibits fibrotic changes in DDC-fed mice. Immunohistochemical staining findings demonstrated that melittin effectively suppresses the expression of FSP-1. Representative immunohistochemical images from each study group (five mice per group) (**A**) NC, normal control group; (**B**) Mel, melittin (0.1 mg/kg)-treated group with normal diet; (**C**) DDC, 0.1% DDC-supplemented diet group; (**D**) DDC + Mel, melittin (0.1 mg/kg)-treated group with 0.1% DDC-supplemented diet; magnification × 200; (**E**) morphometric assessment of the trichrome staining positive areas. Results are expressed as the mean ± SE of three independent determinations. * *p* < 0.05 compared to the NC group. **^†^** *p* < 0.05 compared to the Mel group. **^‡^** *p* < 0.05 compared to the DDC group.

**Figure 8:**
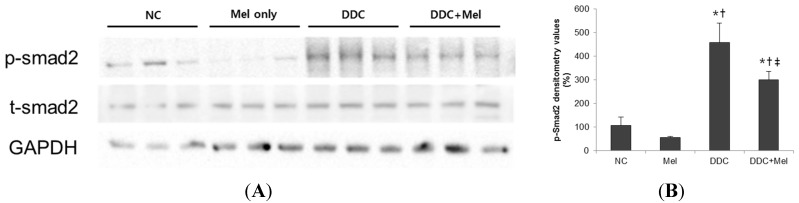
Melittin inhibits phosphorylation of Smad2 in DDC-fed mice. (**A**) Western blotting results demonstrated that melittin effectively suppresses the expression of p-Smad2; (**B**) graphical presentation of the ratio of p-Smad2 to GAPDH in various groups. NC, normal control group; Mel, melittin (0.1 mg/kg)-treated group with normal diet; DDC, 0.1% DDC-supplemented diet group; DDC + Mel, melittin (0.1 mg/kg)-treated group with 0.1% DDC-supplemented diet. Results are expressed as the mean ± SE of three independent determinations. * *p* < 0.05 compared to the NC group. **^†^** *p* < 0.05 compared to the Mel group. **^‡^** *p* < 0.05 compared to the DDC group.

Chronic exposure to injuries in cholangitis becomes a progressive course of cholestatic liver with inflammation and fibrosis of bile ducts. Much attention has been focused on the central role of TGF-β1 upregulation as a prototypical fibrogenic cytokine in liver fibrosis [17]. Western blotting results showed that the expression of TGF-β1 was significantly increased in DDC mice, whereas melittin treatment markedly decreased the expression of TGF-β1 in DDC + Mel livers (Figure 6). The expressions of TGF-β1-regulated ECM protein, fibronectin and vimentin were increased in DDC-fed mice. Treatment with melittin effectively abrogated this increase in the DDC + Mel group. During tissue remodelling in liver fibrosis, FSP-1 is considered a marker of fibroblasts in fibrotic liver. DDC feeding significantly increased the number of cells positive for FSP-1 (Figure 7). However, melittin treatment resulted in a reduction in the FSP-1-positive cells in DDC + Mel livers. Along with the upregulation of TGF-β1 and ECM proteins, the expression level of p-Smad2 was increased by chronic DDC feeding in the DDC group (Figure 8). Melittin treatment attenuated the expression of p-Smad2 in the DDC + Mel group. Taken together, these results show that melittin might protect liver during DDC feeding by attenuating fibrotic gene expression.

### 2.4. Melittin Effectively Suppresses Proliferating Cholangiocytes in DDC-Fed Mice

**Figure 9:**
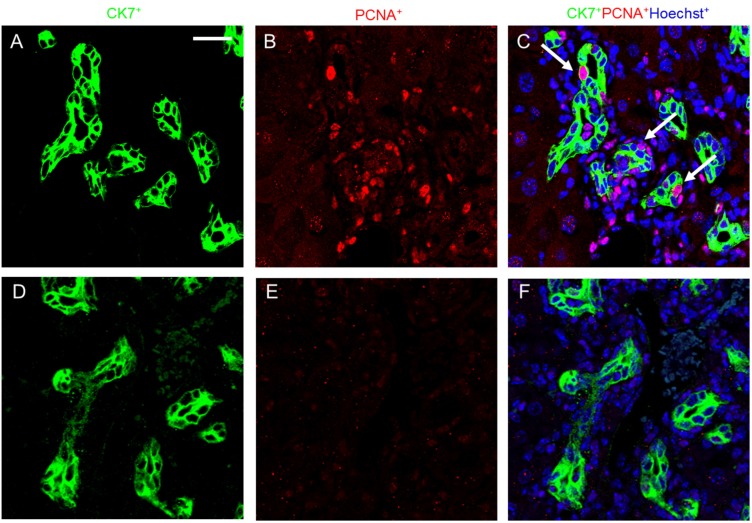
Melittin inhibits cholangiocyte proliferation in DDC-fed mice. Immunofluorescence staining shows co-localization of PCNA staining with CK-7 (arrow head) following DDC treatment. Immunofluorescence staining results demonstrated that melittin effectively suppresses the expression of PCNA. CK-7 and PCNA immune complexes were detected by anti-mouse FITC (green) and anti-rabbit Texas red (red). Nuclei were counterstained with Hoechst 33342 (blue). Representative immunofluorescence staining images from each study group ((**A**–**C**) DDC group; (**D**–**F**) DDC + Mel group). Scale bar = 20 μm.

Proliferating cholangiocytes are characteristic for DDC-fed mice and are a source of growth factors, chemokines, cytokines and other soluble factors. Cholangiocytes as specialized epithelial cells that line the biliary tree are considered as pace markers of biliary fibrosis [18]. Cholangiocyte-specific markers, including CK-7, are expressed in the epithelial cells of bile ductules in enlarged portal tracts. Chronic DDC feeding resulted in a comparable amount of ductular reaction and an increased number of CK-7- and PCNA-positive cells (Figure 9). In contrast, melittin treatment significantly suppressed the proliferation of cholangiocytes, which was shown as the expression of PCNA-positive cells in DDC + Mel livers. In summary, these data proved the ability of melittin to suppress cholangiocyte proliferation in response of DDC-induced liver injury and liver fibrosis.

## 3. Discussion

The bulk of the liver is occupied by parenchymal hepatocytes, but little is known about the physiology of cholangiocytes. Cholangiocytes are epithelial cells that line the biliary system and make up 3%–5% of the liver cell population. The major function of cholangiocytes is closely related to bile flow. The ability of cholangiocytes to proliferate is important in many different human pathological liver conditions that involve this cell type, and those conditions are referred to as cholangiopathies. Pro-inflammatory cytokines may be critically involved in the pathophysiology of several cholangiopathies [19,20]. Thus, this study aimed at investigating the specific effects of melittin on the pathogenesis of DDC-induced sclerosing cholangitis and biliary-type liver fibrosis.

Melittin is a major component of bee venom, which makes up 50%–60% of the dry weight of bee venom. It has been studied for its antibacterial, antiviral and anti-inflammatory properties in various cell types [21]. A low concentration of bee venom has been reported to protect TNF-α/actinomycin d-induced hepatocyte apoptosis [22]. Although melittin has lytic effects on biological and cell membranes when inserted into the phospholipid layer at a high concentration, a concentration of melittin lower than 2 μM does not disrupt the cell membrane [21,23,24]. During the progression of atherosclerosis and restenosis, melittin inhibited aortic vascular smooth muscle cell proliferation by suppressing NF-κB, Akt activation and the mitogen-activated protein kinase pathway [25]. Recently, melittin effectively suppressed skin inflammation in the *P. acnes*-induced *in vitro* and *in vivo* inflammatory models [26]. However, there have been no reports on the effects of *in vivo* cholangiopathy-associated molecular mechanisms of melittin. The current study confirmed the anti-inflammatory function of melittin as an effective inhibitor of inflammatory cytokines and biliary fibrosis. This study demonstrated that melittin reduced the ductular reaction in DDC-induced liver injury, suggesting a potential therapeutic use of this compound in the treatment of cholangitis-related liver fibrosis.

Xenobiotics, which are foreign to an organism, include chemical compounds, detergents and pharmaceuticals. Liver plays a major role in the detoxification and elimination of xenobiotics. DDC feeding is widely used to study xenobiotic-induced liver injury. DDC-induced liver injury is associated with chronic cholestatic liver diseases, which are further related to the induction of a reactive phenotype of bile duct epithelial cells with the development of bile duct injury, leading to portal-portal fibrosis and large duct disease [8,27].

To further evaluate the function of melittin in liver fibrosis, this study used a DDC-induced liver fibrosis model. Chronic DDC feeding elevated the hepatic inflammatory responses in portal fields and hepatocyte injuries and increased collagen deposition and periductal portal-portal fibrosis. Increased serum AST and ALT are known to be major risk factors related to the development of chronic liver disease. Especially, the elevations of serum AP and bilirubin are well-known parameters for cholestatic liver disease. The reductions of serum AP and bilirubin play a major role in mediating the repression of biliary disease [28,29]. Chronic DDC feeding in mice increased the serum levels of AST, ALT, AP and bilirubin. Of particular interest, this study showed that treatment with melittin appeared to decrease AP and bilirubin concentrations in the serum of DDC-fed mice. Elevations of serum AST, ALT, AP and bilirubin, from liver metabolic disorder, play important roles in the initiation of liver fibrosis, and liver metabolic disorder was affected by pro-inflammatory cytokines [30].

Pro-inflammatory cytokines and chemokines have an important role in the initiation and perpetuation of various types of liver fibrosis. Although a previous study has demonstrated that melittin has anti-inflammatory and anti-fibrotic activity in thioacetamide-induced liver fibrosis [31], the precise mechanisms of action of melittin in cholangiopathies remain to be elucidated. TNF-α is a key molecule in the hepatic inflammatory response, the execution of apoptosis and the regulation of liver regeneration. Moreover, the TNF superfamily may represent major players in the immunobiology of sclerosing cholangitis and associated biliary fibrosis [20,32]. A recent study showed that genetic loss of TNFR1 significantly affects the pathogenesis of DDC-induced sclerosing cholangitis and ductular reaction. Consistent with these results, DDC-induced injury led to increased production of TNF-α expression. Along with TNF-α upregulation, the expressions of IL-6, p-STAT3 and MCP-1 were also increased in DDC-fed mice. STAT3 is the major downstream signaling molecule of IL-6 in hepatocytes. The hepatoprotective function of STAT3 in the liver has been well documented in many murine models. Especially, conditionally-inactivated STAT3 in hepatocytes and cholangiocytes led to strongly aggravated hepatocellular damage and fibrosis in a sclerosing cholangitis animal model using mice lacking the multidrug resistance gene 2 (mdr2^−/−^) [33]. Our present study showed that melittin effectively suppressed the expressions of TNF-α, IL-6, p-STAT3 and MCP-1 in DDC-fed mice. These results demonstrate that melittin mediates the anti-inflammatory effect during the resolution of biliary fibrosis in liver.

During liver injury, periportal hepatocytes are damaged, and their proliferation is impaired. Damaged liver parenchyma become a source of regenerating hepatocytes, biliary epithelial cells and draining ductules in order to restore the functional liver mass [34,35]. When liver parenchyma is damaged, the ductular reaction progresses as an alternative pathway for liver restoration. Ductular reaction has been regarded as a barometer of portal fibrosis, since proliferating biliary epithelial cells are a source of molecules that mobilize ECM deposition and secrete pro-inflammatory cytokines and chemokines, which further activate hepatic stellate cells and portal fibroblast [36,37]. This remodelling process of liver milieu demonstrates a strong capacity to increase myofibroblast proliferation and ECM deposition, thus contributing to the fibrogenic response to liver injury [38]. Our current study investigated the question of whether melittin could affect ductular reaction during chronic liver injuries. Chronic DDC feeding led to increased ductular reaction at the portal tract interface, including small biliary ductules with bile plugs and inflammatory cells. In addition, CK-7, which is a cholangiocyte-specific epithelial cell marker, was increased in ductular reaction near the portal tract in DDC-fed mice. Furthermore, proliferating cholangiocytes were also increased by chronic DDC feeding. However, melittin treatment withdrew ductular reactions and cholangiocyte proliferation in DDC-fed mice.

Several studies have demonstrated that proliferating cholangiocytes secrete profibrogenic factors. During biliary fibrosis, proliferating bile duct epithelial cells are the predominant source of the profibrogenic gene, such as connective tissue growth factor (CTGF) [39]. Moreover, epithelial cells of newly-formed bile ducts express mRNA for α1 (IV) procollagen, suggesting that proliferating cholangiocytes are a source of hepatic collagen during fibrosis [40]. The expression of TGF-β1, which is a key upstream signaling molecule of CTGF and the major fibrogenic cytokine in liver fibrosis, was increased in chronic DDC-induced liver injury. Following TGF-β1 upregulation, the expressions of fibronectin, vimentin, FSP-1 (a key marker of early fibroblast lineage) and p-Smad2 were also increased in DDC-fed mice. In contrast, melittin markedly reduced the responses induced by DDC in mice. In fact, melittin treatment downregulated the expression of matrix components during biliary fibrosis.

Overall, this study demonstrated the protective effects of melittin on DDC-induced biliary fibrosis *in vivo*. Treatment of melittin markedly decreased the expression of inflammatory cytokines in DDC-fed mice. Melittin also suppressed biliary fibrosis by attenuating the expression of fibrogenic cytokines and ECM proteins. Moreover, melittin modulated ductular reaction and subsequent fibrosis. These results collectively suggest that melittin may regulate the remodelling process, which involves crosstalk between mesenchymal cells and cholangiocytes in biliary fibrosis. In summary, these findings expand the role for melittin in regulating the expression of ECM and begin to provide insight into its regulatory involvement in liver fibrosis-related pathologies.

## 4. Experimental Section

### 4.1. Animal Model

For the induction of liver injury, 6–8-week-old C57BL/6 male mice (20–25 g; Samtako, Kyungki do, Korea) were used. Mice were fed with a 0.1% DDC-supplemented diets for 4 weeks, housed with a 12-h light/dark cycle and permitted *ad libitum* consumption of water. All animal protocols were approved by the Institutional Animal Care and Use Committee of Catholic University of Daegu (Daegu, Korea). Mice were randomly divided into four groups as follows: (1) untreated group (normal control, NC); (2) melittin-treated group with normal diet (Mel); (3) 0.1% DDC-supplemented diet group (DDC); (4) melittin-treated group with 0.1% DDC-supplemented diet (DDC + Mel). The Mel only group and the DDC + Mel group received intraperitoneal injection of melittin (0.1 mg/kg; Sigma-Aldrich, St. Louis, MO, USA) dissolved in saline twice a week. Mice were sacrificed after 4 weeks of treatment, and the livers were removed.

### 4.2. Histopathology and Immunohistochemistry

Small pieces of liver from each lobe were kept in 10% formalin solution. Paraffin-embedded liver tissues were sectioned and stained with H&E and Masson’s trichrome according to the standard protocol. For immunohistochemistry, sections were incubated with anti-monocyte chemoattractant protein (MCP)-1 and anti-fibroblast specific protein (FSP)-1. After three serial washes with phosphate-buffered saline (PBS), the sections were processed by an indirect immunoperoxidase technique using a commercial kit (LSAB kit; Dako, Glostrup, Denmark). The slides were examined with an Eclipse 80i microscope (Nikon, Tokyo, Japan) and analysed with iSolution DT software (IMT i-Solution, Coquitlam, BC, Canada).

### 4.3. Serum Biochemical Analysis

Serum samples were stored at −70 °C until analysed using a QuantiChrom™ kit (BioAssay Systems, Atlanta, GA, USA) for alanine transaminase (ALT), aspartate aminotransferase (AST), alkaline phosphatase (AP) and bilirubin.

### 4.4. Western Blot Analysis

Liver tissues were homogenized in radioimmunoprecipitation assay (RIPA) buffer (Cell Signaling Technology, Danvers, MA, USA) for 15 min on ice and centrifuged at 12,000 rpm for 15 min at 4 °C. The supernatant was collected, and the residual protein concentration was measured by the Bradford protein assay (Bio-Rad Laboratories, Berkeley, CA, USA). Sodium dodecyl sulphate polyacrylamide gel electrophoresis was performed with 8%–12% polyacrylamide gels at 100 V for three hours. The resolved proteins were transferred from the gel onto a polyvinylidene fluoride (PVDF) membrane (Millipore Corporation, Bedford, MA, USA) and probed with anti-TNF-α, anti-fibronectin, anti-IL-6 (Abcam, Cambridge, UK), anti-t-Smad 2 (Santa Cruz Biotechnology, Dallas, TX, USA), anti-TGF-β1 (R&D Systems, Minneapolis, MN, USA), anti-fibronectin, anti-vimentin (BD Biosciences, San Jose, CA, USA), anti-p-Smad2 (Novus Biologicals, Littleton, CO, USA), anti-p-signal transducer and activator of transcription (STAT)3 and anti-glyceraldehyde-3-phosphate dehydrogenase (GAPDH) (Cell Signaling, Danvers, MA, USA), followed by secondary antibody conjugated to horseradish peroxidase (1:2000) and detected with enhanced chemiluminescence reagents (Amersham Bioscience, Piscataway, NJ, USA). Signal intensity was quantified by an image analyser (Las3000; Fuji, Tokyo, Japan).

### 4.5. Immunofluorescence Staining and Confocal Microscopy

Paraffin-embedded mouse liver sections (3-μm thickness) were prepared by a routine procedure. After blocking with 10% donkey serum for 30 min, the slides were immunostained with primary antibodies against proliferating cell nuclear antigen (PCNA, Santa Cruz Biotechnology, Dallas, TX, USA) and cytokeratin-(CK)-7 (Millipore, Darmstadt, Germany). To visualize the primary antibodies, sections were stained with secondary antibodies conjugated with FITC or Texas red (Invitrogen, Carlsbad, CA, USA). Sections were then counterstained with Hoechst 33342. Stained slides were viewed under a Nikon A1 microscope equipped with a digital camera (Nikon, Tokyo, Japan).

### 4.6. Statistical Analyses

Data are presented as the mean ± SE. Student’s *t*-test was used to assess the significance of independent experiments. The criterion *p* < 0.05 was used to determine statistical significance.

## 5. Conclusions

These data expand the role for melittin in regulating the expression of ECM and begin to provide insight into its regulation by liver fibrosis and related pathologies, PSC and biliary fibrosis.
